# Evaluation of Methods to Collect Diurnal Culicidae (Diptera) at Canopy and Ground Strata, in the Atlantic Forest Biome

**DOI:** 10.3390/insects13020202

**Published:** 2022-02-16

**Authors:** Juliana Telles de Deus, Luís Filipe Mucci, Simone Lucheta Reginatto, Mariza Pereira, Eduardo Sterlino Bergo, Vera Lucia Fonseca de Camargo-Neves

**Affiliations:** Secretaria de Estado da Saúde de São Paulo, Superintendência de Controle de Endemias—Sucen, São Paulo 01027-000, SP, Brazil; julianatd@hotmail.com (J.T.d.D.); lfmucci@gmail.com (L.F.M.); simone_reginato@yahoo.com.br (S.L.R.); marizap222@gmail.com (M.P.); edusteber@uol.com.br (E.S.B.)

**Keywords:** electric trap, insect nets, attractants/semiochemicals, CO_2_, BG-Lure^®^, acrodendrophilic mosquitoes, Culicidae

## Abstract

**Simple Summary:**

Automatic traps employing CO_2_ chemical attractants and BG-Lure^®^ (Biogents AG, Regensburg, Germany) were evaluated as an alternative to insect nets, which is the standard method in Brazil for capturing mosquitoes that transmit sylvatic arboviruses. The collections were conducted during the day, in the forest canopy and ground strata, at an environmental reserve in the Atlantic Forest. From the 18 collections conducted, 3570 specimens from 52 taxa were obtained. Nets were the best way to capture insects. The yield was similar in traps with CO_2_ and traps using CO_2_ combined with BG-Lure^®^. CO_2_ traps can complement collections with nets; however, for species of epidemiological interest in the genera *Haemagogus* and *Sabethes,* insect nets are still the best method, especially in the canopy.

**Abstract:**

Hand-held insect nets are the standard method for capturing vector mosquitoes of sylvatic arboviruses; however, occupational risks and biases due to individual skill and attractiveness are important limitations. The use of chemical attractants and automatic traps could be an alternative to resolve these limitations. This study compares the yields achieved using nets with those employing electrical traps with CO_2_ and BG-Lure^®^, near the ground and in the canopy strata (6.0 and 8.0 m high). The study was conducted at the Cantareira State Park, which is in the Brazilian Atlantic Forest biome. In the 18 collections performed, 3570 specimens of 52 taxa were obtained. The most frequent species captured near the ground were *Wyeomyia confusa* and *Limatus durhamii,* whereas *Sabethes albiprivus, Sabethes purpureus,* and *Haemagogus leucocelaenus* were the most frequent in the canopy. The nets resulted in greater species richness and abundance, followed by the trap employing CO_2_. The combination of CO_2_ traps with BG-Lure^®^ did not improve performance. The use of BG-Lure^®^ alone resulted in low abundance and a low number of species. Our results demonstrate that the use of traps with CO_2_ can be complementary to collections with nets; however, for species of epidemiological interest such as those of the genera *Haemagogus* and *Sabethes*, especially in the canopy, the net remains the method of choice.

## 1. Introduction

A variety of techniques and equipment for entomological collection are used to survey and monitor dipterans of medical interest [1,2]. The choice of the appropriate method must consider specific characteristics of the target species, based on different oviposition, dispersion, rest, shelter and feeding habits, as well as their way of life, seasonality, period of nictemeral activity, and environmental niche [1,2,3].

Collection techniques with human baits for anthropophilic species are considered standard techniques to capture adult mosquitoes and are recommended by the Brazilian Ministry of Health for surveillance and control of sylvatic arboviruses [4]. However, collector bias and occupational risk (exposure to infections, accidents, and adverse environmental factors) are important limitations that must be considered in research and work routines [5]. Occupational risk is especially important when considering work in tree canopies, which is necessary due to the stratification of vector species in Brazilian tropical forests [6].

The use of synthetic attractants may be an alternative approach to reduce the risks associated with entomological captures. Additionally, they may reduce collector bias due to individual skills and intrinsic attractiveness. In Brazil, most studies using synthetic attractants have focused on urban environments and aimed to capture *Aedes aegypti* and *Culex quinquefasciatus* [7,8,9]. For diurnal vectors, carbon dioxide and other synthetic attractants, including octenol and lactic acid, have been used [10,11,12].

To capture nocturnal insects, bait traps with electric lights are employed and, as an alternative to light attraction, chemical attractants can be used to increase their sensitivity, to attract species with diurnal habits in the absence of human attractants [13].

This study assessed the sylvatic Culicidae fauna in the Atlantic Forest biome, by collecting mosquitoes of epidemiological importance in different forest strata (ground and canopy) and to compare traps employing different attractants with the standard technique used in yellow fever surveillance, which consists of mobile capture with hand-held insect nets using humans as attractants.

## 2. Materials and Methods

### 2.1. Study Area

**Figure 1:**
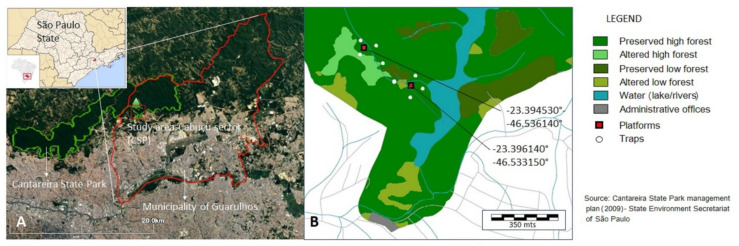
(**A**) Google Earth image (accessed on 30 July 2021)—limits of the Cantareira State Park (green), limits of the municipality of Guarulhos (red); study site (red square). (**B**) Thematic map, adapted from the Cantareira State Park Management Plan (2009), showing the platforms (red squares), the automatic trap installation sites (white dots), and the preserved vegetation belts of different tree heights (different shades of green).

The Cantareira State Park (CSP) is one of the environmental conservation units in the Green Belt of the City of São Paulo established to protect the remnants of the Atlantic Forest biome and the water sources of the main water supply system for the Metropolitan Region of São Paulo. Within its 90.5 km perimeter, the CSP encompasses 7916.52 hectares distributed between the municipalities of São Paulo, Guarulhos, Mairiporã, and Caieiras (Figure 1). The area is divided into four administrative sections where public visitation is allowed, and the annual attendance is approximately 60,000 people. The semideciduous plateau forest has a heterogeneous vegetation cover, due to different stages of regeneration due to variations in soil and terrain, which provide diverse niches for an estimated more than 1600 species of fauna and flora. The climate in the region is classified as mesothermal and humid with a rainy summer and dry winter (Koeppen: Cwa). The average monthly highs are around 23 °C and absolute maximum temperatures can exceed 34 °C; the average monthly lows are around 16.5 °C and absolute minimum temperatures usually reach 9 °C. The rainy season extends from October to March, with an average annual rainfall of 1322 mm [14].

The Cabuçu section was selected for the study. This section is located in the municipality of Guarulhos (Figure 1) and is 2619.4 hectares, with varying degrees of preservation and some developed areas, including a reservoir, operational and administrative buildings, access roads, and sidewalks [14]. Two collection sites were selected, one in a forest area with taller trees and rugged topography and the other in a more heterogeneous landscape near the banks of the Cabuçu Reservoir, with 360 m between them (Figure 1).

### 2.2. Entomological Capture

Entomological collections were conducted during two seasons—spring and summer—from October 2019 to March 2020, which are considered the most favorable to capture Culicidae. Collections occurred on three consecutive days each month, from 10:00 a.m. to 3:00 p.m., using two collection techniques, (1) hand-held nets and oral aspirator with mobile human bait and (2) automatic CDC-type traps [15] with volatile chemical baits.

The two collection areas were defined with the central point by installing the platforms for captures in the canopy stratum (C) (Figure 1B). The first platform was 6.0 m high, and the second 8.0 m high.

Net collections were carried out simultaneously in the canopy (C) and near the ground (G) by two collectors, respectively. Although one collector remained in the canopy, the other covered the area around the platform, covering a radius of approximately 150 m. They alternated the strata in the morning and the afternoon.

Automatic electrical traps (CDC type), powered by a 12V battery, used two chemical attractants: carbon dioxide (dry ice) and Biogents AG (Regensburg, Germany) BG-Lure^®^ attractant (commercial product), composed of ammonia, L-lactic acid, and acid caproic.

In each collection area, eight traps were placed, four traps about 1.5 m above the ground, and another four in the canopy, at a minimum height of 6.0 m and a maximum of 12.0 m, depending on the height of the arboreal stratum of the area. The traps were opened during the same time as the collection with nets took place and were arranged in the north, south, east, and west positions, with the tree containing the platform as the central reference and at least 100 m from it (Figure 1B). Thus, four canopy (C)—ground (G) pairs were defined per collection area, with a pair of traps with only dry ice (CO_2_), called GCO_2_ and CCO_2_; another with just BG-Lure^®^, called GBG and CBG. The other two with both semiochemicals, in diametrically opposite positions, were called GCO_2_ + BG1 and CCO_2_ + BG1, and the second pair, GCO_2_ + BG_2_ and CCO_2_ + BG2. Each day, the pairs of traps were rotated between the cardinal points, so that all the attractants were presented in all directions, to minimize exposure bias.

### 2.3. Registration and Conservation of Samples

The collected insects were transferred to cryotubes using a Castro-type oral aspirator. Then, the mosquitoes stored in cryotubes were killed by freezing them in liquid nitrogen. In the laboratory, the samples were kept in a freezer at −80 °C. Each cryotube received a label containing information about the stratum, collection date, start and end time, and the sample number. A standardized form was used for field records.

### 2.4. Taxonomic Identification

The specimens were identified on a cold table at −20 °C, with the aid of a stereoscopic microscope. Taxonomic keys for Culicidae [2,16,17] were used to identify the genus and species. *Haemagogus janthinomys* and *Hg. capricorii*, whose females are morphologically indistinguishable [2], were grouped as *Hg. janthinomys/capricornii*. Only insects of the Culicidae family were identified. Other Diptera and insects of other orders were discarded.

### 2.5. Data Analysis

To estimate curves related to diversity data, the statistical package EstimateS version 9 [18] was used. For richness (S), the number of new species added to each collection, in each stratum, and the capture technique with the different treatments were considered. Collection efficiency was evaluated using Coleman rarefaction curves, with 100 randomizations, and the estimate of the true number of species was given by the CHAO1 estimator. The average number of singletons (species collected at only one time) and uniques (species that with only a single specimen) per stratum were obtained by the statistical package EstimateS v. 9 [18].

The statistical package PAST version 4.05 [19] was employed to calculate the Shannon’s diversity indices (H), equitability index (J), and Simpson dominance (D) [20]. To calculation the indices, the individuals that could be identified down to the species level were considered, except for the genus *Johnbelkinia*, in which the two specimens captured were considered for the analysis of richness, diversity, and similarity as a single species.

Spearman’s correlation was used for the pairwise comparison of the employed methodology (net and electric trap with the different attractants) and the capture stratum. Bray–Curtis hierarchical clustering analysis by similarity (cluster) was employed to assess the similarity between treatments according to stratum, considering the abundance of all specimens and the species of greatest epidemiological importance. For both analyses, the statistical package PAST version 4.05 [19] was used.

## 3. Results

In the 18 collections carried out, a total of 3575 specimens were obtained, of which 5 specimens were male (Table S1), and due to the small number of males collected, they were not included in the analyses.

The 3570 (99.86%) females collected were distributed in 52 different taxa of the Culicidae family, with 43 identified to the species level, from 12 genera. The most frequent species near the ground were *Wyeomyia confusa* (53.1%) and *Limatus durhamii* (30.9%). *Sabethes albiprivus* (14.5%), *Sabethes purpureus* (13.1%), and *Haemagogus leucocelaenus* (11.3%) were those most frequently captured in the canopy stratum. Additionally, 13 species had only one or two identified specimens, considering all techniques (treatments), in both strata. In total, 6 species were found only in the canopy and 13 species only on the ground, corresponding to rare or not very abundant species.

More mosquito specimens were obtained on the ground (n = 2855) than in the canopy stratum (n = 715) (Table 1). The net technique achieved greater yield than automatic traps, both near the ground and in the canopy, with 63.6% of the total specimens collected in the canopy and 47.3% near the ground. In the canopy, traps using CO_2_ captured a greater abundance, followed by the combination CO_2_ + BGL. For the ground stratum, the set of traps containing CO_2_ + BGL obtained similar numbers as traps with only CO_2_. Traps using exclusively BGL attractant achieved the lowest yield in both strata (Table 1).

The species richness was similar between the canopy and the ground stratum (Table 2). However, the canopy presented greater diversity and equitability than the ground, as well as a lower dominance. The same pattern was obtained when comparing each trap treatment between the strata. The difference in abundance between strata was four times greater at ground level than in canopy considering all methods employed, except for the traps using BGL where abundance was similar in both strata (Table 1).

Among the techniques, the netting obtained the greatest richness in both strata. The diversity, dominance, and equitability indices were similar for the traps with only CO_2_ and the CO_2_ + BGL combination. The BGL traps achieve very different indices from the other treatments in both strata.

**Figure 2:**
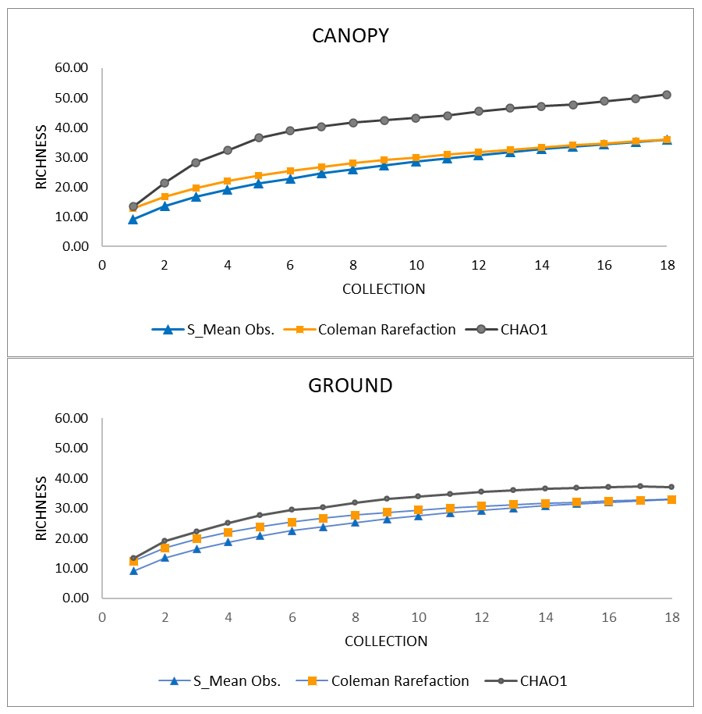
Species accumulation curves of females collected from October 2019 to March 2020, in the canopy and ground strata. Guarulhos, SP, Brazil.

The species accumulation curves (S_Obs) for the two strata displayed a similar pattern, with a tendency to stabilize and follow the estimated curves (Figure 2). When comparing the number of species collected according to stratum with the CHAO1 estimator, the ground had greater sampling efficiency (88.8%) than the canopy (70.5%). The singletons and uniques found near the ground were eight (24.2% of the species collected on the ground) and five (15.5%), respectively, and in the canopy, fourteen (38.8% of the species collected on the canopy) and eleven (30.5%), respectively.

**Figure 3:**
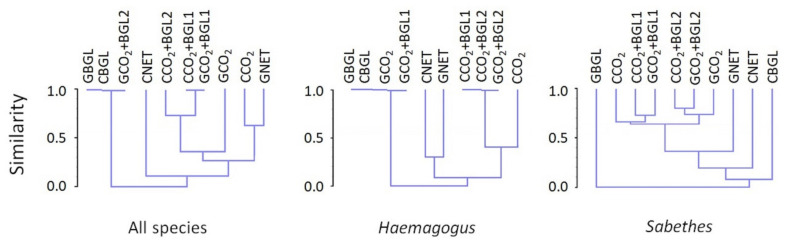
Hierarchical clustering with all female species and genus *Haemagogus* and *Sabethes* according to the method used and stratum. Note: CO_2_ = CDC-like electrical trap using carbon dioxide; BGL = electric trap using the chemical attractant BG-Lure^®^; CO_2_ + BGL (1,2) = two pairs of electrical traps using carbon dioxide and the commercial chemical attractant BG-Lure^®^; NET = hand-held insect nets.

The first dendrogram, with all collected species, has five branches: (1) formed by traps using BGL, in the canopy and near the ground, and CO_2_ + BGL2 near the ground (100% similarity); (2)—Net, in the canopy; (3) CO_2_ + BGL2, in the canopy and CO_2_ + BGL1, in the canopy and near the ground (this group with 70% similarity); (4) CO_2_ near the ground; and (5) CO_2_ in the canopy and net near the ground (with approximately 62% similarity) (Figure 3). The genus *Haemagogus* (Figure 3) had 100% similarity for the branch with BGL, captured in the canopy and near the ground with GCO_2_ and GCO_2_ + BGL1 and, for the CCO_2_ + BGL1,2 and GCO_2_ + BGL2 branch. For the genus *Sabethes* (Figure 3), the similarity was 65% for all treatments, except for BGL and net, both methods in the canopy and on the ground (Figure 3).

The comparison between the different methodologies and strata is in Table 3. The methods with the strongest correlation were pair of traps employing CO_2_ in the canopy compared with traps with both attractants CO_2_ + BGL1 (ρ = 0.84), CO_2_ + GBGL2 (ρ = 0.79), in the canopy and on the ground, respectively, and for the net (ρ = 0.79) on the ground. In the canopy, a moderate correlation was observed with the pairs CO_2_ and CO_2_ + GBGL2 (0.62), CO_2_ and CO_2_ + CNET (ρ = 0.68). On the ground, the CO_2_ trap had a significant correlation with CO_2_ + BGL1 in the canopy (ρ = 0.64). A moderate correlation was observed with traps using CO_2_ + BGL1,2 in the canopy with the same treatment near the ground (ρ = 0.61 and ρ = 0.62, respectively) and a high correlation with the pairs CO_2_ + BGL, in the canopy and CO_2_ + BGL2, near the ground (ρ = 0.86). The combinations using traps with BGL did not have a significant correlation with hand-held net or traps treated with CO_2_ and CO_2_ + BGL (1,2) both in the canopy and near the ground (data not shown).

## 4. Discussion

The CSP is part of the ‘Green Belt of the City of São Paulo’ and contains a preserved forest with a high degree of connectivity to other large forest fragments in the Metropolitan Region of São Paulo [14]. Knowledge of the Culicidae fauna and other groups of pathogen vectors in this area is strategic for epidemiological surveillance in these cities, as they are some of the most populous in the country, with a total of approximately 21.6 million inhabitants. Consequently, both the public use of this environmental protection area and the extremely urbanized occupation of its surroundings can pose a significant health threat.

No other entomological studies have been conducted in the Cabuçu section of the CSP. Therefore, this study increases the knowledge of the Culicidae fauna of the park as a whole, especially the species with diurnal activity, which include 11 species of the Aedini tribe and 31 species of the Sabethini tribe, mainly the genera *Wyeomyia* and *Trichoprosopon*, frequently found in the preserved dense ombrophilous forest of the Serra do Mar [21,22,23,24]. The period evaluated—spring and summer—is especially favorable for the development of immature forms of these groups due to the frequent rains that fill natural breeding sites, including tree hollows, bamboo internodes, and leaf axils [2].

The attractants used in our study, regardless of the method employed, were aimed at capturing females, which were 99.86% of the captured and analyzed specimens.

*Wyeomyia confusa* and *Limatus durhamii* were the dominant species, corroborating recent findings in other sections in the CSP, where several capture techniques were applied for adults and immature forms [23,24]. Ceretti-Júnior et al. (2020) [23] conducted captures in two other sections of the CSP, located in the cities of São Paulo and Mairiporã. They identified 88 Culicidae species, distributed in 16 genera, of which only 23 species coincided with those identified in this study, and the greatest difference was in the *Culex* genus. Montes (2005) [24] studied two areas in those municipalities, employing automatic traps using the CO_2_ attractant, and collected 24 species of Culicidae, of which only 10 coincided with those collected in this study in the Cabuçu section, in Guarulhos. This difference can be explained in part by the selectivity of day verses the night collection. Some species of medical importance, such as *Haemagogus janthinomys/capricornii, Psorophora albigenu, Sabethes albiprivus,* and *Sa. belisarioi* found in this study, were not observed by these two authors [23,24].

In this study, both the selection of the spring and summer seasons and the diurnal period limited the complete survey of the Culicidae fauna. However, these periods were suitable for collecting the target species, mainly from the Aedini tribe (especially the genus *Haemagogus*) as well as those of the genus *Sabethes*.

Most of the pathogen-transmitting mosquito species collected in the Cabuçu section are associated with the yellow fever arbovirus, which significantly affected the population of howler monkeys (*Alouatta* sp.) in the CSP, between 2017 and 2018 [25,26]. Although *Haemagogus janthinomys/capricornii* and *Hg. leucocelaenus* are considered the main vectors in Brazil [27], natural infections have been reported in *Ae. albopictus, Ae. scapularis*, *Ae. serratus, Ps. albipes, Ps. Ferox,* and *Sa. albiprivus*, [27,28,29,30,31,32,33,34], and experimental infection has confirmed *Ae. aegypti* [34]. *Aedes aegypti* and *Ae. albopictus,* species associated with urban arboviruses, such as dengue (DENV), Zika (ZIKV), and chikungunya (CHIKV) in the country [35,36], were collected in low abundance in the Cabuçu section, which was expected in the sylvatic environment.

Analyses of species accumulation and biodiversity allowed evaluation of sampling sufficiency, description of different richness between the strata and the collection techniques, and evaluation of the various treatments with different attractants in electric traps. The accumulation curves tended to stabilize for both strata, indicating that the collection effort was satisfactory. However, the high numbers of singleton and doubletons in the canopy suggest that further samplingmau reveal more rare species. When comparing the observed richness to that obtained by the CHAO1 estimator, a higher sampling efficiency was obtained near the ground (88.8%) than in the canopy (70.5%), probably because the latter stratum contained lower abundance. In addition, the majority of species collected there had less than 10 individuals and with a large proportion were rare species, represented by singletons and uniques, which were more frequent in the canopy than near the ground. Nevertheless, a comparative analysis can be made between collection techniques and attractants, especially with species of greater epidemiological importance (*Haemagogus janthinomys/capricornii* and *Hg. leucocelaenus)*. These species, compared with other studies, were relatively more abundant [37,38].

The obtained diversity and dominance results can be mostly attributed to the greater abundance near the ground of *Wyeomyia confusa* and *Limatus durhami*, which together accounted for 69.9% of the specimens (Table 1). The dominance of these species also affected the diversity and equitability indices when comparing strata. The diversity indicator was higher in the canopy (H = 2.64) than near the ground (H = 1.32) in the different methods (Table 2). Confalonieri and Costa Neto (2012) [39] observed the opposite in their study conducted in the Caxiuanã forest of the state of Pará, where they found greater diversity in ground collections and a decrease in diversity indices at other heights. These authors performed collections in both day and night periods, which may explain the difference in the diversity from that found in this study. Hendy et al. (2020) [40] did not observe differences in diversity between different vertical stratifications of mosquitoes in the Amazonforest associated with microclimate variations.

In general, cluster analysis indicated low similarity between the traps using BGL and the other treatments, which is due to the low abundance and richness found. The CO_2_ bait was more effective alone than in combination with BGL, and their combined use did not seem to have a synergistic effect when compared with traps with only CO_2_. The nets using humans as bait in the canopy were the least similar to the other treatments and were the most effective method. The net method stood out, especially for the targeted species that vector the yellow fever virus. In general, depending on the set of attractants used, greater similarity between groups with different treatments was observed for the *Sabethes* genus and the different strata for the *Haemagogus* genus.

The use of BG-Lure^®^ both in the canopy and near the ground did not result in greater abundance or improve the efficiency of the electric traps, and this combination did not correlate with any of the other attractants (CO_2_ and human), which corroborates other authors, who studied Neotropical sylvatic daytime mosquitoes [40]. This indicates the need to develop in situ studies with olfactometry and different attractants and dosages for mosquitoes in the Neotropical region. On the other hand, the positive correlations and high similarity of the CO_2_ trap in the canopy with the other treatments in both strata, as observed in the dendrograms, suggest a possible alternative to the netting technique with humans as bait. In addition, traps employing CO_2_ could complement the netting technique in some cases. However, because obtaining dry ice in rural and sylvatic areas is not easy, both operationally and logistically, the use of CO_2_ containers with a pressure gauge regulated flow might work better, but flow and cost analyses are also necessary.

Improving the performance of electric traps has been the subject of several studies with chemical attractants [11,41,42] or with the exposure of animals as bait [43]. In this study, using the combination of traps with the chemical attractants CO_2_, BG-Lure^®^, and CO_2_ + BG-Lure^®^, led to greater abundance for the CO_2_ traps, and the richness was similar to that of the CO_2_ + BG-Lure^®^ combination. Although the BG-Lure^®^ attractant was used according to the manufacturer’s instructions, its use alone resulted in low abundance and low richness, and its combination with CO_2_ did not increase the sensitivity of the trap. Other authors obtained similar results [41,44] when they used traps with attractants based on octenol compared with CO_2_ traps.

In the evaluation of the collection methods, a greater richness was found using the insect net, in both strata, with 16 species of mosquitoes collected using only this method (36% of the total). The net also proved to be more effective for sampling *Hg. leucocelaenus* and *Hg. janthinomys/capricornii,* which reinforces the importance of human bait when collecting Culicids, compared with those carried out with traps employing attractants. Nevertheless, as discussed above, traps with only CO_2_ in the canopy provided good results and deserve further investigations with this attractant.

Despite the operational difficulties of the work in the tree canopy, the results demonstrate the relevance of collections in this stratum, where 84.2% of female specimens of the genus *Haemagogus* and 66.3% of specimens of the genus *Sabethes* were obtained. In this research, both *Hg. janthinomys/capricornii* and *Hg. leucocelaenus* were predominant in the canopy of trees, and in the worked areas their height varied between 6 and 12 m. Pinto et al. [39] studied the mosquito fauna in different forest strata in the Amazon region, where trees reach 30 m in height, and found the predominance of *Hg. janthinomys* in the canopy and *Hg. leucocelaenus* on the ground. Hendy et al. (2020) [40] also observed the vertical stratification of *Hg. janthinomys* and *Sa. chloropterus*, possibly due to significant differences in relative humidity and temperature between the ground and other strata. Despite the marked acrodendrophilic behavior of *Hg. leucocelaenus*, its presence near the ground has also been reported [32,37,38,39]. In our study, we observed a greater presence of this species on the ground using net collection, probably due to the active collection of catchers who walked along the trails on the perimeter of each platform, causing a forest intrusion effect.

Entomological surveillance of emerging arboviruses, such as yellow fever in the Atlantic Forest, is an important activity aimed at obtaining the greatest possible number of vector mosquito species, considering the hourly variations and different capture strata, as well as the need for capture methods complementary to only those that use humans as attractants. Using synthetic attractants in electrical traps should be a complementary method. Our results showed, in the studied area in the Atlantic Forest biome, it is not yet possible to replace the hand netting method with a human attractant for target species which transmit the Amaryllic virus and other arboviruses, especially at the canopy level. However, our results using different methods and attractants allowed the capture of greater richness of important species for surveillance of emerging arboviruses. In addition to monitoring the richness and abundance of these target species, for an efficient surveillance system, complementary studies on natural infection by these viruses and on feeding habits of these mosquitoes are still necessary. This study highlights the importance of periodic monitoring with different capture methods and attractants, such as humans and CO_2_, by public health surveillance services.

## 5. Conclusions

The collections in the canopy of trees in the Atlantic Forest biome resulted in a greater richness of diurnal mosquitoes of medical importance, mainly with the use of a human collector equipped with a hand-held net, which is still the recommended method to obtain species that transmit yellow fever. The data showed that the electric trap, originally designed for night use with light bait, is efficient to obtain diurnal mosquito species when employing CO_2_. Thus, they are indicated as a complementary collection method to human catchers equipped with nets, especially in the canopy. The treatment of traps with BG-Lure^®^ was not effective for capturing species of diurnal mosquitoes from the Atlantic Forest, requiring further studies in different biomes, such as the Cerrado, in areas with records of yellow fever cases and other arboviruses. Other analyses related to the feeding habits of these species, as well as the natural infection, should still be considered in relation to the method employed.

## Figures and Tables

**Table 1:** Number of female specimens collected by genus and species, according to stratum (canopy and ground) and capture technique (treatment) between October 2019 and March 2020. Guarulhos, SP, Brazil.

TAXA	CANOPY							GROUND							TOTAL	
	BGL	CO_2_	CO_2_ + BGL1	CO_2_ + BGL2	NET	TOTAL	%	BGL	CO_2_	CO_2_ + BGL1	CO_2_ + BGL2	NET	TOTAL	%	N	%
*Aedes aegypti*			1		2	3	0.4		1	1		1	3	0.1	**6**	**0.2**
*Aedes albopictus*					1	1	0.1					1	1	0.0	**2**	**0.1**
*Aedes scapularis*		1				1	0.1		1	2		2	5	0.2	**6**	**0.2**
*Aedes terrens*							0.0					4	4	0.1	**4**	**0.1**
*Aedes* sp.							0.0	1				1	2	0.1	**2**	**0.1**
*Anopheles (K) homunculus*					1	1	0.1							0.0	**1**	**0.0**
*Culex quinquefasciatus*		4	2			6	0.8		2			1	3	0.1	**9**	**0.3**
*Culex* sp.		1			3	4	0.6		6	2		7	15	0.5	**19**	**0.5**
*Haemagogus janthinomys/capricornii*					53	53	7.4					5	5	0.2	**58**	**1.6**
*Haemagogus leucocelaenus*		4	1	1	75	81	**11.3**				1	17	18	0.6	**99**	**2.8**
*Haemagogus* sp.					1	1	0.1							0.0	**1**	**0.0**
*Johnbelkinia* sp.		1				1	0.1				1		1	0.0	**2**	**0.1**
*Limatus durhamii*	4	25	5	4	13	51	7.1	2	181	178	226	294	881	**30.9**	**932**	**26.1**
*Psorophora albigenu*					1	1	0.1							0.0	**1**	**0.0**
*Psorophora albipes*							0.0					3	3	0.1	**3**	**0.1**
*Psorophora ferox*					2	2	0.3		2		1	11	14	0.5	**16**	**0.4**
*Psorophora lutzii*							0.0					1	1	0.0	**1**	**0.0**
*Psorophora* sp.		1				1	0.1		1				1	0.0	**2**	**0.1**
*Runchomyia perturbans*			1			1	0.1							0.0	**1**	**0.0**
*Runchomyia theobaldi*					1	1	0.1			1	1	2	4	0.1	**5**	**0.1**
*Runchomyia* sp.	1	1	1		4	7	1.0			2		4	6	0.2	**13**	**0.4**
*Sabethes albiprivus*		14	6	3	81	104	**14.5**		1		3	18	22	0.8	**126**	**3.5**
*Sabethes aurescens*		9	13	8	20	50	7.0		8	12	8	38	66	2.3	**116**	**3.2**
*Sabethes belisarioi*					5	5	0.7							0.0	**5**	**0.1**
*Sabethes identicus*	5	1		2	4	12	1.7		1	1	1	23	26	0.9	**38**	**1.1**
*Sabethes imperfectus*			1			1	0.1		1			2	3	0.1	**4**	**0.1**
*Sabethes intermedius*		3	2	4	13	22	3.1		6	3	3	6	18	0.6	**40**	**1.1**
*Sabethes purpureus*		2	2	2	88	94	**13.1**			1		2	3	0.1	**97**	**2.7**
*Sabethes undosus*					3	3	0.4							0.0	**3**	**0.1**
*Sabethes whitmani*				5	2	7	1.0				1		1	0.0	**8**	**0.2**
*Sabethes* sp.		3	2	3	4	12	1.7		6	3	3	8	20	0.7	**32**	**0.9**
*Shannoniana fluviatilis*	2	3		1	1	7	1.0		10	3	4	53	70	2.5	**77**	**2.2**
*Trichoprosopon compressum*							0.0		2		2		4	0.1	**4**	**0.1**
*Trichoprosopon digitatum*					2	2	0.3		2	2		4	8	0.3	**10**	**0.3**
*Trichoprosopon magnum*							0.0					1	1	0.0	**1**	**0.0**
*Trichoprosopon pallidiventer*			3	1		4	0.6	1	20	14	4	9	48	1.7	**52**	**1.5**
*Trichoprosopon pallidiventer/castroi/simile*							0.0			6	2		8	0.3	**8**	**0.2**
*Trichoprosopon walcotti*		6				6	0.8			3	9	2	14	0.5	**20**	**0.6**
*Trichoprosopon* sp.		3	4		2	9	1.3		6	2	9	6	23	0.8	**32**	**0.9**
*Wyeomyia caracula*							0.0					2	2	0.1	**2**	**0.1**
*Wyeomyia confusa*	6	21	8	4	9	48	6.7	11	230	204	262	808	1515	**53.1**	**1563**	**43.8**
*Wyeomyia lateralis*					2	2	0.3							0.0	**2**	**0.1**
*Wyeomyia limai*		8	3	1	4	16	2.2		2		1		3	0.1	**19**	**0.5**
*Wyeomyia longirostris*					1	1	0.1							0.0	**1**	**0.0**
*Wyeomyia lutzi*				1	3	4	0.6					2	2	0.1	**6**	**0.2**
*Wyeomyia oblita*				1		1	0.1							0.0	**1**	**0.0**
*Wyeomyia pallidoventer*			1			1	0.1							0.0	**1**	**0.0**
*Wyeomyia palmata*					2	2	0.3							0.0	**2**	**0.1**
*Wyeomyia personata*							0.0					2	2	0.1	**2**	**0.1**
*Wyeomyia serrata*		4	1	1	11	17	2.4					4	4	0.1	**21**	**0.6**
*Wyeomyia theobaldi*					6	6	0.8							0.0	**6**	**0.2**
*Wyeomyia* sp.	1	11	7	9	35	63	8.8		3	4	2	16	25	0.9	**88**	**2.5**
**TOTAL**	**19**	**126**	**64**	**51**	**455**	**715**	**100.0**	**15**	**492**	**444**	**544**	**1360**	**2855**	**100.0**	**3570**	**100.0**
**%**	2.7	17.6	9.0	7.1	63.6	100.0		0.5	17.2	15.6	19.1	47.6	100.0			

Note: CO_2_ = CDC-like electrical trap using carbon dioxide; BGL = electric trap using the chemical attractant BG-Lure^®^; CO_2_ + BGL (1,2) = two pairs of electrical traps using carbon dioxide and the commercial chemical attractant BG-Lure^®^; NET = hand-held insect nets.

**Table 2:** Biodiversity indices according to stratum and capture method by total females collected. Guarulhos, SP, Brazil.

STRATUM	CANOPY						GROUND					
METODOLOGY	BGL	CO_2_	CO_2_ + BGL1	CO_2_ + BGL2	NET	TOTAL	BGL	CO_2_	CO_2_ + BGL1	CO_2_ + BGL2	NET	TOTAL
Taxa_S	4	15	15	15	27	36	3	16	14	17	28	33
Individuals	17	106	50	39	406	618	14	470	431	530	1318	2763
Shannon_H’	1.32	2.28	2.32	2.45	2.31	2.64	0.66	1.24	1.20	1.10	1.34	1.32
Dominance_D	0.28	0.13	0.13	0.11	0.14	0.10	0.64	0.39	0.40	0.43	0.43	0.40
Equitability_J	0.95	0.84	0.86	0.90	0.70	0.74	0.60	0.45	0.45	0.39	0.40	0.38

Note: CO_2_ = CDC-like electrical trap using carbon dioxide; BGL = electric trap using the chemical attractant BG-Lure^®^; CO_2_ + BGL (1,2) = two pairs of electrical traps using carbon dioxide and the commercial chemical attractant BG-Lure^®^; NET = hand-held insect nets.

**Table 3:** Comparison of different capture and stratum methods (C = canopy and G = ground). Spearman correlation coefficients (ρ) and statistical significance values (*p*) presented for significant pairs.

Stratum/Method			ρ	*p*
CCO_2_	x	CCO_2_ + BGL1	0.84	0.0012
CCO_2_	x	CCO_2_ + BGL2	0.62	0.0413
CCO_2_	x	CNET	0.68	0.0221
CCO_2_	x	GCO_2_ + BGL2	0.79	0.0042
CCO_2_	x	GNET	0.79	0.0037
CCO_2_ + BGL1	x	GCO_2_	0.64	0.0342
CCO_2_ + BGL1	x	GCO_2_ + BGL2	0.61	0.0448
CCO_2_ + BGL2	x	GCO_2_ + BGL1	0.62	0.0405
CCO_2_ + BGL2	x	GCO_2_ + BGL2	0.86	0.0007

Note: CO_2_ = CDC-like electrical trap using carbon dioxide; BGL = electric trap using the chemical attractant BG-Lure^®^; CO_2_ + BGL (1,2) = two pairs of electrical traps using carbon dioxide and the commercial chemical attractant BG-Lure^®^; NET = hand-held insect nets.

## Data Availability

The study did not report any data.

## Supplementary Materials

The following supporting information can be downloaded at: https://www.mdpi.com/article/10.3390/insects13020202/s1: Table S1: Number of male specimens collected by genus and species.
