# Supplementary material for: Evaluation of Methods to Collect Diurnal Culicidae (Diptera) at Canopy and Ground Strata, in the Atlantic Forest Biome
Source: Insects. 2022 Feb 16;13(2):202. doi: 10.3390/insects13020202 (PMC8874964; doi:10.3390/insects13020202)
Supplement: Supplementary file 1 [file insects-13-00202-s001.zip › insects-1509252-supplementary.pdf]

Table S1.—Number of male specimens collected by genus and species, according to stratum (canopy and ground) and capture technique (treatment) between October 2019 and March 2020. Guarulhos, SP, Brazil.

Specie	N	Method	Stratum
Culex sp	2	net	ground
Haemagogus janthinomys/capricornii	1	net	canopy
Aedes aegypti	1	net	canopy
Trichoprosopon pallidiventer	1	CO_2*	ground
TOTAL	5		

* Note: CO_2 = CDC-like electrical trap using carbon dioxide.
